# Target-Based Physiological Modulations and Chloroplast Proteome Reveals a Drought Resilient Rootstock in Okra (*Abelmoschus esculentus*) Genotypes

**DOI:** 10.3390/ijms222312996

**Published:** 2021-11-30

**Authors:** Kaukab Razi, Dong-Won Bae, Sowbiya Muneer

**Affiliations:** 1 Horticulture and Molecular Physiology Lab, School of Agricultural Innovations and Advanced Learning, Vellore Institute of Technology, Vellore 632014, India; kaukab.razi@vit.ac.in; 2 School of Biosciences and Technology, Vellore Institute of Technology, Vellore 632014, India; 3 Central Instrument Facility, Gyeongsang National University, Jinju 52828, Korea; bdwon@gnu.ac.kr

**Keywords:** *Abelmoschus esculentus*, chloroplast proteome, drought stress, physiological modulations, resilient rootstock

## Abstract

As climate changes increase, drought stress is becoming a problem for all major horticultural crops; among them is okra (*Abelmoschus esculentus*). Despite its superior resilience to heat stress and high nutritional content, it is still underutilized in contrast to other vegetable crops. Moreover, the drought-resistant and drought-sensitive genotypes of okra are also not well known and require further exploration to improve their productivity. To investigate this in more detail, we performed comparative physiological and large-scale chloroplast proteomics on drought-stressed genotypes of okra. We evaluated four major genotypes of okra, viz., NS7774, NS7772, Green Gold, and OH3312 for drought resilient rootstock. The physiological modulations demonstrated a significant change by 50–76% in biomass, net-photosynthetic machinery, water transport, and absorption both in early and late stages of drought stress compared to well-watered crops in all genotypes. Maximum oxidative damage due to drought stress was observed for the genotypes NS7772, Green Gold and OH3312 as depicted by H_2_O_2_ and O_2_^−^ determination. Greater oxidative stress was correlated to lesser antioxidant activity and expression of antioxidant enzymes, such as catalase and ascorbate peroxidase under stress in okra genotypes. The overall photosynthetic pigments, such as total chlorophyll, and total carotenoid content, were also decreased, and stomatal guard cells were disrupted and appeared closed compared to the control for the above three mentioned genotypes, except NS7774. A subsequent tissue-specific proteome analysis of chloroplasts and thylakoids analyzed by BN-PAGE (blue native polyacrylamide gel electrophoresis) revealed either over or under expression of specific proteins, such as ATPase, PSI, PSII core dimer, PSII monomer and ATP synthase. The expression of multiprotein complex proteins, including PSII-core dimer and PSII-core monomer, was slightly higher for the genotype NS7774 when compared to three other genotypes for both 5 and 10 days of drought stress. Further identification of specific proteins obtained in second dimension BN-PAGE provided descriptive detail of seven proteins involved in drought resistance across all genotypes. The identified proteins are majorly involved in photosynthesis under drought stress, suggesting NS7774 as a drought tolerant genotype. Further, the proteomic results were confirmed using Immunoblot by selecting specific protein such as PsaA. Overall, from our physiological modulations and chloroplast proteomics in all genotypes, we summarized NS7774 as a resilient rootstock and the other three genotypes (NS7772, OH3312, and Green Gold) as sensitive ones.

## 1. Introduction

Drought stress is one of the major environmental stresses that affects plant growth, survival, and production [1,2]. Even a small percentage reduction in plant water supply disrupts photosynthesis, limits metabolic reactions, cuts down the CO_2_ exchange, and causes stress-related damage to chloroplasts [3,4,5]. Every vegetable crop has different drought responses, depending upon the severity of the drought, through the different growing phases of the plants. Under moderate drought stress, the initial response of plants includes poor germination [6,7,8]. Whereas severe drought stress results in impaired growth of the plants and reduced biomass, height, leaf size, and stem girth [9,10,11]. In addition to the morphological aspects, physiological performances, such as photosynthetic rate, stomatal movements, and chlorophyll fluorescence, are also affected significantly under drought stress [8,9,10,11,12,13]. Consequently, the chloroplast, which is the organelle responsible for photosynthesis, becomes affected, because it is highly sensitive to drought stress, which results in a generation of more reactive oxygen species (ROS), decreasing the chlorophyll synthesis, quantum yield, and efficiency of light-harvesting complexes (LHC) I and II, which ultimately decrease the health and viability of the plant [14]. It has been studied that the chloroplast provides a defense mechanism to plants by providing various metabolites, which are produced during photosynthesis. However, it is very important to study the effect of drought stress on the chloroplast proteome of vegetables to provide solutions for improved plant growth.

Furthermore, accumulation of reactive oxygen species (ROS) is found in plants under several abiotic stresses, including drought stress, which leads to oxidative stress and the malfunctioning of cells by damaging lipids and proteins [13,15]. Disturbance in the biochemical activity is another effect on plants, whereby malondialdehyde (MDA) levels and relative water content (RWC) of leaves are altered under drought stress, leading to lipid peroxidation [16,17]. Plants adapt to stresses due to antioxidant defense mechanisms to counteract oxidative stress, such as catalase activity (CAT) and superoxide dismutase (SOD), and ascorbate peroxidase activity (APX) is incremented under drought stress as a defense mechanism [18]. Several studies have been conducted regarding the function of these defensive enzymes and how they help plants to develop tolerance under drought stress [18]. However, the mechanism behind stress response behaves differently in every crop, either improving their resistance or making them tolerant towards stress conditions. Moreover, there are limited studies on important and underutilized vegetable crops on their stress resilience. A detailed study on important vegetable crops has to be investigated in order to identify stress signaling pathways and homeostatic maintenance under abiotic stress. Investigating the resilient rootstock mechanism of crop’s physiological modulations is one of the important traits to take into consideration. The proteomic approach can be used for further research studies as an ideal tool to elucidate the underlying mechanism of crops’ resistance to abiotic stresses, including drought. Several studies have been conducted using proteomic tools for plants under drought stress, including tomatoes [8], wheat [19], and Arabidopsis [20]. However, only a limited number of specific thylakoidal proteomic studies have been conducted on vegetable crops, such as okra (*Abelmoschus esculentus*), to understand the mechanism behind stress resilience.

During 2015–2016, it was estimated that vegetables occupied a major portion of the horticulture sector, approximately an area of 9.6 million ha out of a total production of 166.6 million tones, and an average productivity of 17.4 tons/ha in India, constituting 59% of horticulture production in India [21]. Okra (*Abelmoschus esculentus* L.) is one of the important vegetable crops grown in warm regions of India, because of its high economic and nutritious value. This vegetable is known for its high content of vitamins, folic acid, carbohydrates, calcium, potassium, and other minerals, which are of great value for the human diet [22]. In addition to that, okra seeds are used to generate a particular oil, which is highly essential for human nutrition. Considering all these important benefits of okra, it has become very important to grow these crops on a large scale [23]. However, as we know, the cultivation of this vegetable is increasing with time, so it is very important to maintain proper irrigation levels and water availability as drought stress can be a major problem for this crop [24]. Moreover, okra is a common vegetable crop grown in southern parts of India and yet is the most underutilized crop. A number of genotypes are being used to grow this crop, but limited information is known about the resilient rootstocks of this particular crop, particularly under drought stress. Therefore, the present research has been conducted to reveals a drought resilient rootstock in okra genotypes, focusing on their physiological modulations, and chloroplast and thylakoidal proteomes. Furthermore, the current study was conducted to determine the tolerance and homeostatic pathways behind the selected genotypes for further exploration to increase their yield using various agricultural practices.

## 2. Materials and Methods

### 2.1. Plant Materials and Drought Treatments

Seeds of okra (*Abelmoschus esculentus* L.) genotypes viz., NS7772, NS7774, Green Gold, and OH3312 (widely used in the southern region of India), obtained from Namdhari Seeds Private Limited and Syngenta company Vellore, TN, India, were germinated and grown in pots in the polyhouse of the VIT School of Agricultural Innovations and Advanced Learning (VAIAL). The pots with a caliber of 25 cm × depth 19 cm were filled with a soil composed of red soil, sand (for proper aeration and moisture content), and vermicompost (for proper nutrients) in the ratio of 1:1:1. The seeds were sown at a depth of around 3–4 cm. The temperature was set to 30–35 °C with a relative humidity of 70–90% (monitored with the help of a digital temperature sensor; Richie HTC Infrared/Optical Thermometer). The pH of the soil was maintained at 6.5–7.0 and checked continuously during scheduled irrigation times. During the vegetative period, pots were divided into two groups: one set that was the control, and another set that was induced with drought stress for a period of ten days. Around 40 pots, each with four plants, underwent a drought treatment and 40 were well-watered to evaluate the morphological changes. For physiological and proteomic analysis, five independent biological replicates (five pots with four plants each) were considered for experimental analysis. Drought stress was induced naturally by withholding scheduled irrigation. The rest of the pots were irrigated using drip irrigation. Plants were observed every day, starting from day 1 to day 10 of drought treatment, and water moisture was observed by weighing the pots and by using a soil moisture sensor. For biochemical and proteomic analysis, leaves were harvested on the 5th and 10th days of drought treatment and immediately frozen in liquid N_2_ before being stored in a deep–freezer (−80 °C) for further analysis.

### 2.2. Morphological Measurements

All the potted crops were monitored morphologically and were photographed regularly under controlled and drought-stressed conditions for ten consecutive days of treatment. The morphological parameters, including fresh weight, dry weight, length of root, and shoot were measured carefully using standard protocols. For the fresh weight, the plants were pulled out from pots along with the roots, carefully washed with distilled water, and weighed on a digital weighing balance for accurate measurements. For measuring the length of the root and the shoot standard, measuring scales were used and the data were recorded.

### 2.3. Measurement of Relative Water Content (RWC), MDA Content and Proline Content

All the plants were taken out along with the roots from the soil on the 5th and 10th day of drought stress treatment. The plants were washed carefully, and then weighed on a weighing balance for fresh weight. For turgid weight, all plant samples were placed for about 2 h in separate petri dishes containing distilled water and then were weighed again to measure the turgid weight. For dry weight, the samples were kept in hot air-dry oven at 65 °C for 48 h continuously and were then weighed. The following formula was used after calculating fresh weight, dry weight and turgid weight in order to calculate relative water content. Relative water content (RWC) % = (FW − DW)/(TM − DW) × 100 where FW indicates fresh weight, DW indicates dry weight and TM indicates turgid weight [25].

Malondialdehyde content (MDA) content was performed according to the protocol given by Heath and Packer [26]. For this experiment, one gram of fresh leaf samples was extracted in 0.1% trichloroacetic acid (TCA), and centrifugation was performed at 10,000× *g* for 5 min. Further 0.5% thiobarbituric acid (TBA) was mixed with 1 mL of supernatant taken from the previous step and was heated at 95 °C for 30 min, terminated on ice and centrifuged at 5000 rpm for 5 min. Lastly, the supernatant was collected, and the absorbance was read at 532 nm and corrected for unspecific turbidity after subtraction from the value obtained at 600 nm.

Proline content was determined by Bates et al. [27]. Homogenization of fresh leaf samples (0.5 g) was conducted in 3% aqueous sulfosalicylic acid. Further centrifugation was done at 2000× *g* for 10 min. A mixture containing acid ninhydrin, glacial acetic acid, and 2 mL of enzyme extract was heated for exactly 1 h in a hot water bath. After one hour, the reaction was terminated on an ice bath. Further extraction was conducted with 4 mL of toluene, along with robust mixing using a glass rod or vortex for 10 sec. The absorbance of the mixture was read at 520 nm and toluene was used as blank.

### 2.4. H_2_O_2_ and O_2_^−1^ Localizations

To detect H_2_O_2_ localization, a solution of 1% of 3,3′-diaminobenzidine (DAB) in Tris-HCl buffer (pH 6.5) was prepared and all the fresh leaf samples were immersed in petri plates containing the solution, followed by 5 min vacuum infiltration and then incubation at room temperature for 16 h in the dark condition. Leaves were immersed in ethanol for the bleaching process and kept in a hot water bath at 65 °C till the brown spots appeared, characterizing the reaction of DAB (3,3′-diaminobenzidine) with H_2_O_2_. Photographs were taken using a digital camera. For the O_2_^−^ detection, a 0.1% solution of nitro blue tetrazolium (NBT) was prepared in K-phosphate buffer (pH 6.4), containing 10 mM Na-azide, and fresh leaves were taken and immersed in petri plates containing the above solution. Vacuum-infiltration was conducted for 5 min and illumination was carried out until the dark blue spots appeared (characteristic of blue formazan precipitate). After bleaching in boiling ethanol, the leaf samples were photographed as described above [28].

### 2.5. Antioxidant Enzyme Assays

Sodium phosphate buffer (50 mM) accommodating 0.05% Triton × 100, 2% polyvinylpyrrolidone, 1 mM EDTA was prepared for enzyme extraction for APX and CAT assays. A total of 100 mg of fresh leaf samples were homogenized in the phosphate buffer. After enzyme extraction, centrifugation was performed at 18,000× *g* at 4 °C for 20 min, and the supernatant obtained was taken for enzyme and protein assays. The standard curve was obtained using the Bradford assay and the protein content of the enzyme aliquot was determined. UV-spectrophotometer (Shimadzo UV-1280, Kyoto, Japan) was used to record the absorbance of all enzyme assays.

For determining APX activity, 0.3 g of tissues were homogenized in 3 mL of extraction buffer (KH_2_PO_4_, K_2_HPO_4_, 1% PVP, 1% TritonX100, and EDTA) and 1 mL of 5 mM ascorbate. The samples were centrifuged for 20 min at 10,000 rpm at 4 °C and the resulting supernatant was mixed with 2 mL of reaction buffer containing KH_2_PO_4_, and K_2_HPO_4_ (pH 7.3). The absorbance was determined at 290 nm with 30 s intervals for 3 min (E = 2.8 mM^−1^ cm^−1^) [29].

CAT activity was estimated by using 50 mM phosphate buffer containing 100 μL of enzyme extract and 15 mM H_2_O_2_. Around 0.2 g of plant tissue were homogenized in extraction buffer containing KH_2_PO_4_, K_2_HPO_4_ (pH 7.4), 1% PVP, 1% Triton, and EDTA. The samples were centrifuged for 20 min at 10,000 rpm and resulting supernatant was reacted with reaction buffer (K_2_HPO_4_, KH_2_PO_4_) and H_2_O_2_ as an enzyme substrate. The absorbance was measured at 240 nm for 3 min in 30 s interval (E = 39.4 mM^−1^ cm^−1^) [30], which was used to determine the H_2_O_2_ scavenging activity.

### 2.6. Water Transport Activity

Fresh plant samples were uprooted and washed properly. They were fixed in 0.1% food-coloring agent for 5–6 h and then rinsed with water. The stem of every cultivar was cut into transverse sections with the help of a sharp surgical razor-blade and was observed under a dark field and phase contrast microscope (model: MT4300L, MEIJI TECHNO CO., LTD., Kyoto, Japan) [8,31].

### 2.7. Photosynthetic Measurements

The net photosynthetic rate, transpiration rate, and stomatal conductance were measured using a portable SPAD meter (Konika Minolta, Tokyo, Japan). Another instrument identified as the PAM 2000 chlorophyll fluorescence meter (Heinz Walz GmbH, Zarges 40860, Weilheim, Germany) was used to measure chlorophyll fluorescence (Fv/Fm). For this parameter, the leaves were adapted to dark conditions for 30 min before measurement. The maximum PS II quantum yield (Fv/Fm) was calculated as Fv/Fm = (Fm − F0)/Fm [32].

### 2.8. Pigment Analysis

Total chlorophyll content and carotenoid content were the two photosynthetic pigments determined using dimethyl sulfoxide (DMSO), as described by Hiscox and Israclstam [33]. Fresh leaf samples were weighed and taken in glass vials, along with 10 mL DMSO, and incubated in a hot air oven at 65 °C for one hour, so that all the pigments were leached out of the leaves. After one hour of incubation, once all the pigments were leached out, the absorbance was observed using a UV-VIS spectrophotometer (Shimadzo UV-1280, Kyoto, Japan) at 410, 510, 663, and 445 nm, and recorded accordingly. Pigment content was analyzed by the formulae given by Arnon [34].

### 2.9. Determination of Stomatal Index

For stomatal observation, a thin layer of leaf tissues (outer epidermal layer) was carefully peeled out. It was laid on a glass slide, covered with a coverslip by adding a few drops of water, and observed under a dark field and phase contrast microscope (model: MT4300L, MEIJI TECHNO CO., LTD., Kyoto, Japan) under 10× magnification as described in previous studies [35]. Stomatal index is the percentage of the total number of stomata to the total number of epidermal cells per unit area of a leaf. The stomatal index was calculated by dividing the number of stomata counted by ten times the area of 1 grid square.

### 2.10. Scanning Electron Microscope (SEM) Analysis for the Structure of Stomata

Fresh plant samples were harvested on the 5th and 10th days of treatment, and then the fresh leaves were cut into small pieces for sample preparation to study the stomatal structure. Afterwards, the leaves were fixed in the first fixative solution i.e., glutaraldehyde solution, for 2–3 h with pH 7.4. After the fixation step, the next step that followed was dehydration, in which the samples were dehydrated with an ethanol series ranging from 95% ethanol to 50% ethanol. The next process for visualizing the structure of stomata was performed using scanning electron microscope (model: EVO-18 Research, Carl Zeiss, United States of America) [36].

### 2.11. Total Protein Profile by SDS-PAGE (Sodium Dodecyl Sulphate Polyacrylamide Gel Electrophoresis)

The relative total protein profile was first evaluated in the first dimension using sodium dodecyl polyacrylamide gel electrophoresis (SDS-PAGE) [37]. The plant samples were grinded to fine powder using liquid nitrogen. The extraction of protein was performed using an extraction buffer containing 40 mM (*w*/*v*) Tris-HCl, pH 7.5, 2 mM (*w*/*v*) EDTA, 0.07% (*w*/*v*) β-mercaptoethanol, 2% (*w*/*v*) PVP (polyvinylpyrrolidone) and 1% (*v*/*v*) Triton X-100 followed by centrifugation of the extracts at 13,000× *g* for 10 min at 4 °C. Protein-dye containing 240 mM Tris-HCl (pH 6.8), 40% glycerol, 8% SDS, 0.04% bromophenol blue and 5% beta-mercaptoethanol was mixed with the supernatant obtained after centrifugation. Quantification of protein samples was conducted using the Bradford assay and a standard curve was plotted using BSA (bovine serum albumin) [38]. The protein samples were loaded on 12.5% polyacrylamide gel and the gel was ran and stained later using Coomassie Brilliant Blue (CBB stain).

### 2.12. D BN-SDS-PAGE

Fresh leaves were collected from the leaves of control and drought stress treated plants for both day 5 and day 10 of the stress treatment. Fist dimension BN-PAGE of integral thylakoid proteins was performed according to previous studies [25]. The leaves were carefully washed with distilled water and were immediately grinded to fine powder in a pestle and mortar using liquid nitrogen. Around 5 g of powdered samples were homogenized in a pre-chilled buffer (PH 7.8), constituting a mixture of 330 mM sorbitol/2 mM EDTA/50 mM HEPES/2 mM Na/5 mM MgCl_2_. The mixture was filtered via Mira cloth. Then centrifugation was performed at 4500× *g* for 10 min at 4 degrees and pellets obtained were re-suspended in the same ice-cold buffer. Centrifugation was performed again, following the first step. Then another buffer containing 20 mM tricine, 70 mM sucrose, and 5 mM MgCl_2_ (pH 7.8) was used, and the pellet obtained above was suspended again, followed by centrifugation at 4500× *g* for 10 min at 4 degrees. Afterwards, the washing of the final pellet was performed twice for approximately 2 min each using the washing buffer to obtain a purified protein pellet (330 mM sorbitol, 50 mM Bis-Tris-HCl, pH 7.0, and 0.1 mgmL^−1^ pefabloc). The final pellet obtained after washing was dissolved carefully in 2% *w*/*v* n-dodecyl-ß-D-maltoside for solubilization and further mixed with 0.1% loading dye (5% CBB-G250, 100 mM Bis-Tris-HCl, pH 7.0, 30% *w*/*v* sucrose and 500 mM ε-amino-n-caproic acid). The protein samples extracted from the above steps were loaded on 5–12% *w*/*v* acrylamide gradient gel (1.5 mm). The Bradford assay was used to determine the protein concentration. First dimension BN-PAGE was performed by running the gel electrophoresis at 4 °C in a Protean II xi Cell electrophoresis system (Bio-Rad, Hercules, California, USA) for a constant voltage of 100 volts for 5–6 h until the gel run was complete. Once the first dimension BN PAGE was completed, protein bands were observed, which were further used for second dimension. The lanes were cut out using a sharp razor blade and further SDS sample buffer containing 1% β-mercaptoethanol and sodium dodecyl sulphate (SDS) was prepared for incubating the gel lanes obtained from 1D BNPAGE for 30 min at room temperature. The lanes were overlaid on 12.5% resolving gel and SDS PAGE was ran at a constant voltage of 100 volts using the protean II xi cell electrophoresis system (Bio-Rad, Hercules, CA, USA) for protein separation. The gel was stained with Coomassie Brilliant Blue-R250 for the identification of protein spots.

### 2.13. Image Analysis

Image analysis was performed using a high-resolution digital camera (Nikon) for both the first- and second-dimension gel images. A total of three replicates (lanes) were performed for 1D-BN-PAGE of each treatment.

### 2.14. Protein In-Gel Digestion and Identification by Matrix Assisted Laser Desorption and Ionization Time of Flight Mass Spectrometry (MALDI-TOF-TOF-MS)

The protein spots obtained in Coomassie stained gel from 2D BN PAGE were cut out using a sharp razor blade followed by in-gel digestion using trypsin (Promega). Matrix-assisted laser desorption and ionization time of flight mass spectrometry (MALDI-TOF/TOF-MS) was used for protein identification using MASCOT server (Matrix Science, www.matrixscience.com, accessed on 19 October 2021, London, UK).

### 2.15. Western Blots (Immunoblot)

For western blotting, the above-mentioned protein extraction method was followed for first dimension gel electrophoresis (SDS-PAGE) for all the leaf samples. SDS-PAGE (1D) was performed, the protein gels were transferred to a PVDF membrane (Bio-Rad, Hercules, CA, USA) and western blot was performed according to Muneer et al. [39]. Once the protein was transferred to the membrane, the blocking solution that was prepared using 5% non-fat dry skimmed milk was used to block the blot immediately after the transfer was done, and side by side, another blot, which was Coomassie stained, was used as loading control. Incubation with polyclonal primary antibody of dilution factor 1:1000 of anti PsaA (Agrisera # AS06 172) for PsaA was conducted for 1:30 h. After primary antibody incubation, the bot was incubated for another one hour with a secondary antibody with a 1:1000 dilution factor identified as HRP-linked anti-rabbit 1gG (Cell Signaling #7074). Image analysis was performed using super signal west Pico chemiluminescent substrates (Cell Signaling Signal Fire ECL Reagent #6883) on a ChemiDoc imaging system (Bio-Rad, Hercules, CA, USA).

### 2.16. Statistical Analysis

Statistical analysis was performed using a Statistical Analysis Software (SAS) tool (Cary, NC, USA). A complete randomized design was utilized with five biological replicates, which were expressed as the mean ± SE. Differences among all treatments were determined using a two-way analysis of variance followed by a Student’s *t*-test with *p* < 0.05 as the limit of significance.

## 3. Results

### 3.1. Genotypic Variation on Morphology of Drought-Stressed Okra

**Figure 1:**
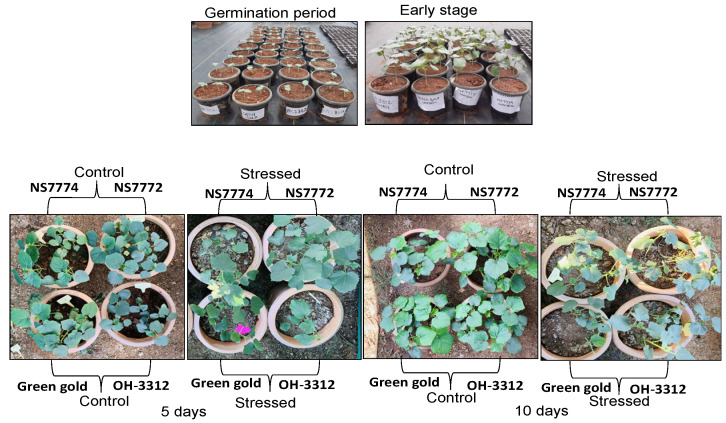
Morphological representation of okra genotypes NS7774, NS7772, Green Gold, and OH3312. Photographs represent the early germination period, early stage and vegetative stage induced by drought stress along with control plants. A total of 5 days represent an early stage, and 10 days represent the late stages of the drought period.

**Figure 2:**
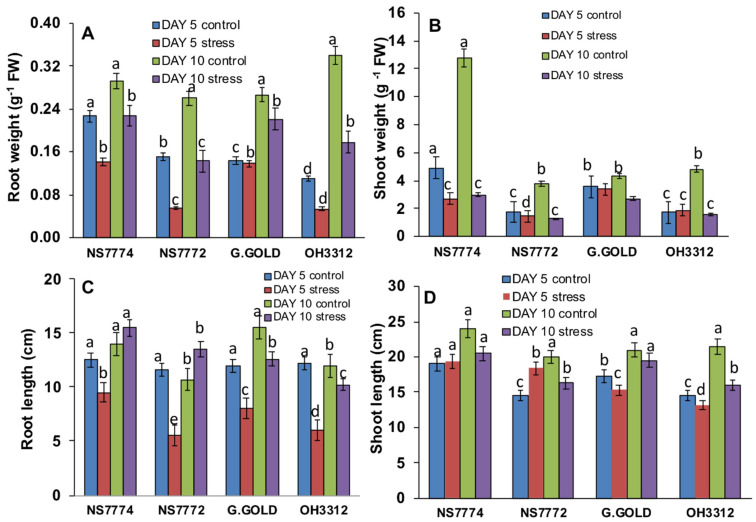
Changes in (**A**) root weight, (**B**) shoot weight, (**C**) root length, and (**D**) shoot length, as affected by drought stress at 5 and 10 days of interval in okra genotypes NS7774, NS7772, Green Gold and OH 3312 as along with their controls. Vertical bars indicate mean ± SE for n = 5. Means denoted by a different letter are significantly different at *p* ≤ 0.05 according to the Tukey’s studentized range test.

Early stages of drought stress (day 5) had little impact on the morphology of all the genotypes of okra (NS7774, NS7772, Green Gold, and OH3312) (Figure 1), whereas significant changes were observed at later stages of drought stress. Early drought stresses brought a significant reduction in all the morphological parameters, including the root and shoot weight as well as root and shoot length. It was noted that most of the morphological attributes were reduced under drought conditions. For both days 5 and 10, the root and shoot weights of the drought-stressed plants were lower than those of the control plants (Figure 2).

Among the four genotypes, the NS7774 cultivar was observed to have a minor reduction in root weight and shoot weight for both moderate and later stages of drought. Similar trends were noted for the root and shoot length for all the okra genotypes. For day 5, there was a decline in root weight for all the cultivars, including NS7774, NS7772, Green Gold and OH3312 by 37–50%. Similarly, root length for day 5 was reduced for all the genotypes by 24–50% compared to the control. Shoot weight was reduced for all genotypes (NS7774, NS7772, Green Gold and OH-3312) on day 5, whereas NS7774 and NS772 had 20% increases in shoot length, respectively. For day 10, a similar trend was followed, in which all the parameters were reduced under drought stress except for the cultivar NS7774, which had the lowest reduction in root weight by −22.1%, shoot weight by −76%, shoot length by −14% and root length by 10%, as compared to the control.

### 3.2. Genotypic Changes in Relative Water, MDA, and Proline Content in Drought-Stressed Okra

**Figure 3:**
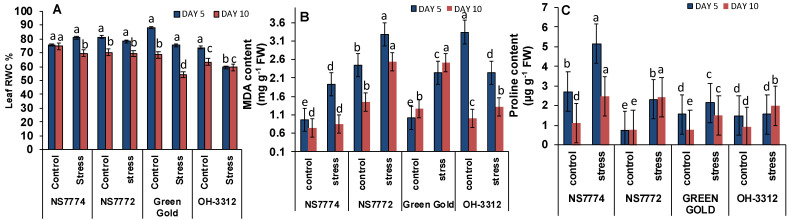
Changes in (**A**) relative water content, (**B**) Malanoaldehyde content, and (**C**) proline content, as affected by drought stress at 5 and 10 days of interval in okra genotypes NS7774, NS7772, Green Gold, and OH3312, along with respective controls. Vertical bars indicate mean ± SE for n = 5. Means denoted by the different letter are significantly different at *p* ≤ 0.05 according to the Tukey’s studentized range test.

Relative water content was reduced on day 5 and gradually decreased further with the increase in drought stress on day 10. For day 5, NS7774 genotype had the highest RWC values by 7% while the remaining three genotypes, NS7772 (−3.6%), Green Gold (−14%), OH-3312 (−19%) had lower RWC as compared to control (Figure 3A). A similar trend was observed on day 10, where RWC decreased in all genotypes of okra viz., NS7774, NS7772, Green Gold and OH-3312, respectively, as compared to their controls.

### 3.3. Oxidative Damage in Drought-Stressed Okra Genotypes

Oxidative damage was observed under drought stress treatments for both day 5 and day 10 as thiobarbituric acid reactive substances (TBARS). When the plants were exposed to drought stress continuously, MDA content accumulated rapidly from day 5 to day 10. On day 5 of drought stress, a significant increase in TBARS was observed for all the okra genotypes viz., NS7774, NS7772, Green Gold and OH3312 by 90% (Figure 3B), as compared to their control, whereas significantly increased MDA content was observed in Green Gold under day 10 drought stress conditions by 100% and the least increment was observed in NS7774 by 12% compared to the control, followed by an increase of 31% in OH3312 and 70% in NS7772. Out of all the genotypes, NS7774 seems to have minimum oxidative damage caused by lipid peroxidation and comes out to be a resilient rootstock.

### 3.4. Proline Content Changes in Drought-Stressed Okra Genotypes

When plants are exposed to drought stress, accumulation of proline content is one of the important indicators to study the plant response. Under day 5 drought stresses, the proline content gradually increased over time for each of the okra genotypes: NS7774, NS7772, Green Gold and OH-3312 by 90%, 21%, 37% and 5%, respectively, as compared to their control values, and cultivar NS7774 had the highest accumulation of proline (Figure 3C). Similarly, for day 10 drought stress condition, an increment in proline content was observed by 128%, 127%, 98%, 111% for the genotypes NS7774, NS7772, Green Gold and OH 3312, respectively, as compared to control levels.

### 3.5. In situ H_2_O_2_ and O_2_^−1^ Localization in Drought-Stressed Okra Genotypes

**Figure 4:**
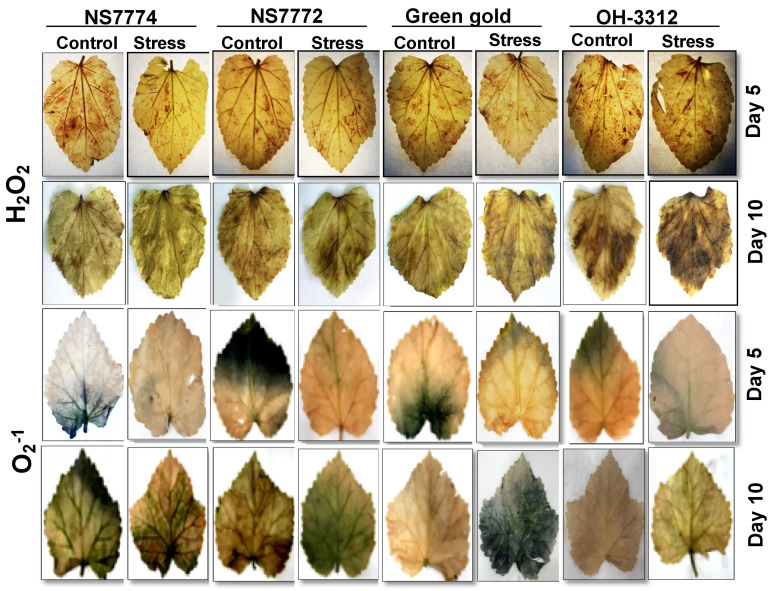
Histochemical localization of oxidative stress markers H_2_O_2_ and O_2_^−^, as affected by drought stress at 5 and 10 days of interval in okra genotypes NS7774, NS7772, Green Gold, and OH3312, along with respective controls. The brownish colour on the leaves indicates the localization of H_2_O_2_ stress marker, whereas the bluish colour indicates the localization of O_2_^−1^ marker.

Histochemical localization was used to detect the accumulation of oxidative stress in the form of H_2_O_2_ and O_2_^−^. The accumulation of H_2_O_2_ was identified by the visualization of brownish colour (as a result of the DAB reaction) on sections of leaf samples, whereas the accumulation of O_2_^−1^ was identified by the presence of dark blue spots because of the blue formazan formed during the NBT reaction (Figure 4).

Under day 5 drought stress, there was brown stain on the leaves of all the okra genotypes, namely NS7774, NS7772, Green Gold and OH 3312, except the cultivar NS7774, which had a lesser stain, showing less oxidative stress as compared to the control. The same pattern of staining was observed following 10 days of treatment for all the cultivars. Under both day 5 and day 10 of drought stress treatment, a few dark blue stains were present over all the leaves of all the genotypes, showing oxidative stress caused by O_2_^−1^ radicle.

### 3.6. Enzyme Activities in Drought-Stressed Okra Genotypes

**Figure 5:**
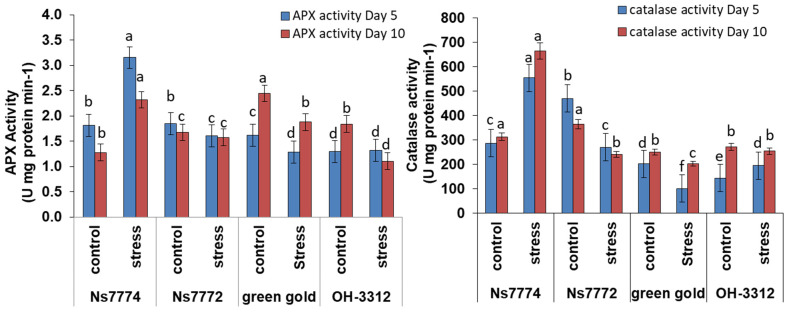
Changes in antioxidant enzyme activity: ascorbate peroxidase (APX) and catalase activity (CAT) activity, as affected by drought stress at 5 and 10 days of interval in okra genotypes NS7774, NS7772, Green Gold, and OH3312, along with respective controls. Vertical bars indicate mean ± SE for n = 5. Means denoted by the different letter are significantly different at *p* ≤ 0.05 according to the Tukey’s studentized range test.

ROS homeostasis is maintained by the activity of antioxidant enzymes produced by plants. Under drought stress conditions, the activities of two antioxidant enzymes viz., catalase (CAT) activity and ascorbate peroxidase (APX) activity, increased to certain folds and gradually kept increasing as the drought stress conditions increased (Figure 5). However, contrasting results were obtained as under day 5 drought stress, CAT activity increased for genotypes NS7774 and OH3312 by 41% and 33%, respectively, as compared to their control, whereas a significant decrease was observed for the other two genotypes, viz., NS7772 and Green Gold, by −40% and 37%, respectively (Figure 5). Similarly, on day 10, the highest catalase activity was for NS7774 by 72%, followed by 10% for OH3312, followed by −32% and −20% for NS7772 and Green Gold, respectively. A similar trend was observed for APX activity, in which NS7774 had the highest amount of APX activity under day 5 and day 10 drought stress condition by 27% and 47%, respectively, as compared to control, whereas for the other three genotypes viz., NS7772, Green Gold and OH 3312, APX activity reduced under drought stress conditions (Figure 5).

### 3.7. Vascular Activity in Drought-Stressed Okra Genotypes

**Figure 6:**
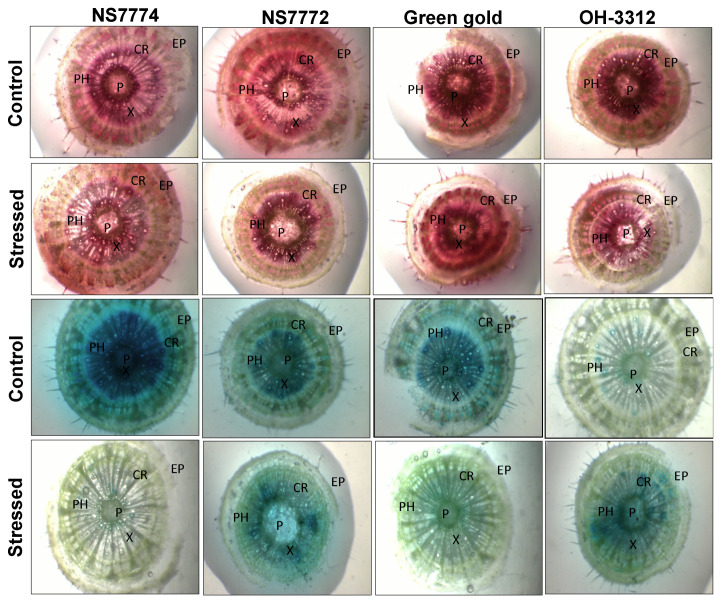
Vascular transport, as affected by drought stress at 5 and 10 days of interval in okra genotypes NS7774, NS7772, Green Gold, and OH3312, along with respective controls. Red and blue food-coloring dyes were used for the absorption process, indicating activity of vascular tissue. EP indicates epidermis; CR indicates cortex; X indicates xylem; P indicates phloem.

A food-coloring dye was used to observe the water transport and vascular activity (xylem and phloem) (Figure 6). At the initial stage of drought stress, a good vascular activity was observed in all okra genotypes due to excessive food-coloring agent observed in all tissues (Figure 6). While at later stages of drought stress, all genotypes showed a possible reduction in vascular activity compared to controlled ones, except for the genotype NS7774, depicting it as a drought resilient genotype compared to other genotypes.

### 3.8. Photosynthetic Changes in Drought-Stressed Okra Genotypes

**Figure 7:**
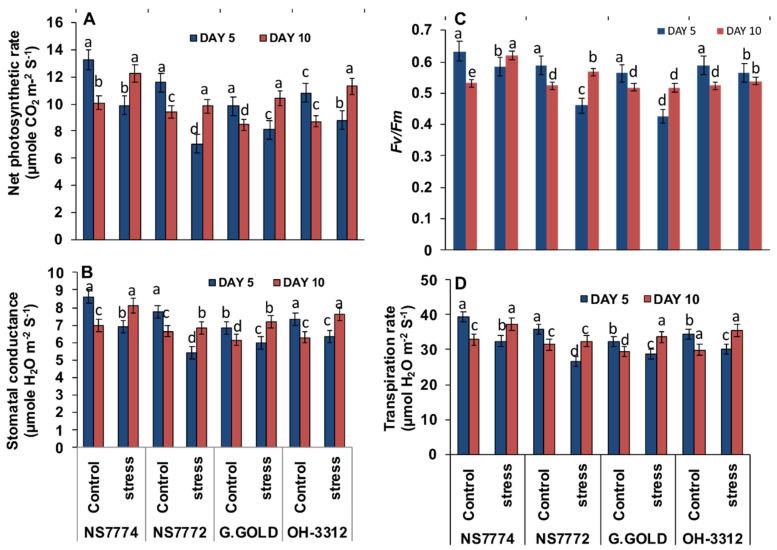
Changes in photosynthetic parameters: (**A**) net photosynthetic rate (**B**) stomatal conductance, (**C**) Fv/Fm, and (**D**) transpiration rate, as affected by drought stress at 5 and 10 days of interval in okra genotypes NS7774, NS7772, Green Gold, and OH3312, along with respective controls. Vertical bars indicate mean ± SE for n = 5. Means denoted by the different letter are significantly different at *p* ≤ 0.05 according to the Tukey’s studentized range test.

The photosynthetic rate, stomatal conductance, and transpiration rate were significantly decreased under the day 5 drought stress condition, whereas they increased under day 10 drought stress. The extent of decrease in these three parameters for day 5 drought stresses was minimum for NS7774 genotype (Figure 7), whereas it was maximum for the day 10 drought stress for the same genotype, showing this cultivar as a more resilient rootstock.

The photosynthetic capacity of a plant is identified by maximum quantum yield, which is the ratio of variable to maximum fluorescence: Fv/Fm. Fv/Fm values for day 5 drought stress treatment were lower for all the genotypes as compared to their control values. Whereas the Fv/Fm values increased for day 10 drought stress treatment for all the genotypes compared to control values (Figure 7C). The NS7774 genotype had the highest value of Fv/Fm under drought stress for 10 days of treatment, depicting it as the most resilient among all selected genotypes.

### 3.9. Changes in Photosynthetic Pigments in Drought-Stressed Okra Genotypes

**Figure 8:**
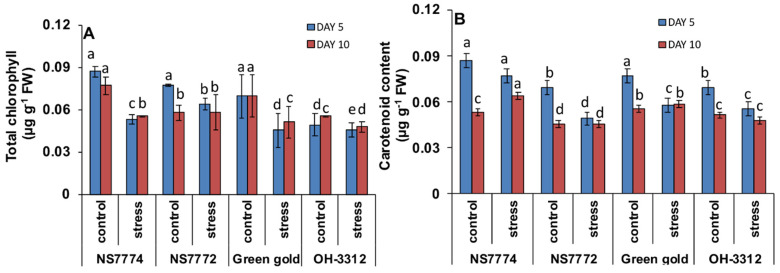
Changes in the photosynthetic pigments: (**A**) total chlorophyll content and (**B**) carotenoid, as affected by drought stress at 5 and 10 days of interval in okra genotypes NS7774, NS7772, Green Gold, and OH3312, along with respective controls. Vertical bars indicate mean ± SE for n = 5. Means denoted by the different letter are significantly different at *p* ≤ 0.05 according to the Tukey’s studentized range test.

All types of photosynthetic pigments, including chlorophyll content and carotenoid content, gradually decreased with an increase in drought stress from day 5 to day 10. At day 5, reduction in the chlorophyll content was observed, with the least reduction in NS7774 by −29% as compared to the control, followed by −16% in Green Gold, −10% in NS7772 and −1% in OH3312. Similarly, on day 10, chlorophyll content was reduced for all four genotypes compared to their control (Figure 8A).

Carotenoid content was reduced on day 5 for all the genotypes, with the least reduction observed in the NS7774 by −59.4% as compared to control. However, under 10 days of drought stress, the okra genotypes viz., NS7774 and Green Gold showed an increase in the carotenoid content compared to the control, whereas NS7772 and OH 3312 showed a decrease in the carotenoid content by −34% and −49%, respectively, as compared to their control, thus showing NS7774 as the best genotype among all of them (Figure 8B).

### 3.10. Stomatal Observations in Drought-Stressed Okra Genotypes

**Figure 9:**
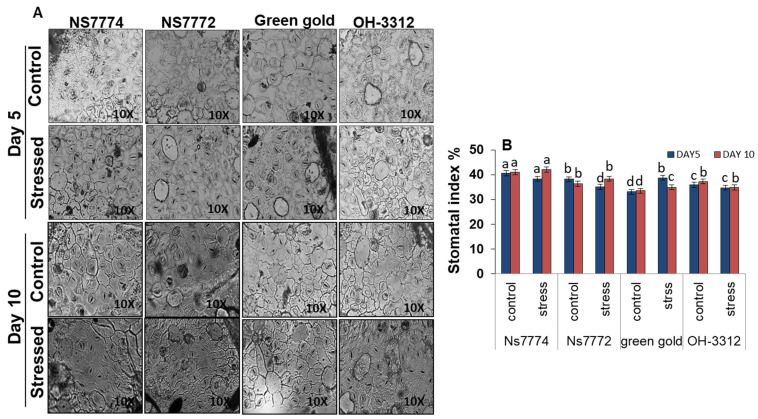
(**A**) Representative image of stomata, and (**B**) stomatal index, as affected by drought stress at 5 and 10 days of interval in okra genotypes NS7774, NS7772, Green Gold, and OH3312, along with respective controls at 10X magnification. Vertical bars indicate mean ± SE for n = 5. Means denoted by the different letter are significantly different at *p* ≤ 0.05 according to the Tukey’s studentized range test.

It was observed that under day 5 and day 10 of drought stress treatment, the number of stomata calculated was less as compared to control plants (Figure 9).

**Figure 10:**
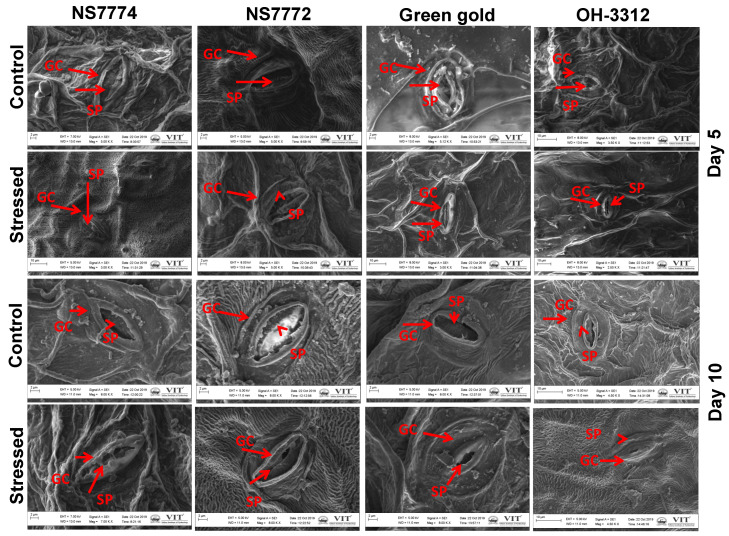
Representative images of stomata, as affected by drought stress at 5 and 10 days of interval in okra genotypes NS7774, NS7772, Green Gold, and OH3312, along with respective controls observed under scanning electron microscope (SEM) at 40X magnification. In images, GC indicates guard cells and SP indicates stomatal pore.

Under 5 days of treatment, closure of stomata was observed but the guard cells remained intact and showed very few significant changes, whereas under 10 days of drought stress treatment, complete closure of stomata along with the guard cells was observed for all the genotypes NS7774, NS7772, Green Gold and OH 3312 (Figure 10). Under 10 days of drought, it was also observed that for genotypes OH3312 and Green Gold, the guard cells were completely damaged (Figure 10).

### 3.11. Proteomic Changes in Drought-Stressed Okra Genotypes

**Figure 11:**
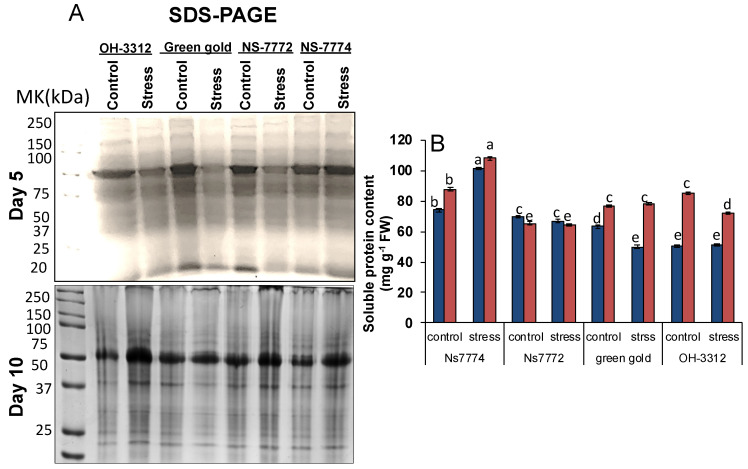
Changes in protein profile (**A**) SDS-PAGE and (**B**) soluble protein content, as affected by drought stress at 5 and 10 days of interval in okra genotypes NS7774, NS7772, Green Gold, and OH3312, along with respective controls. Afterwards, proteins extracted from leaves were run on 12% polyacrylamide gel electrophoresis and stained with Coomassie Brilliant Blue R-250. (Vertical bars indicate mean ± SE for n = 5. Means denoted by the different letter are significantly different at *p* ≤ 0.05 according to the Tukey’s studentized range test.

Initially, the proteome profile was observed using first dimensional SDS-PAGE (Figure 11A) to check the enrichment of proteins for further thylakoidal proteomics. We observed a specific change in proteins in all the genotypes of okra under drought stress. The protein bands were observed to be either up-regulated or down-regulated among all genotypes. At the initial stages of drought stress, the protein expression was observed to be down-regulated while at later stages of drought it was up-regulated. Most distinctly, the abundant protein RuBisCo (Ribulose-1,5-bisphosphate carboxylase/oxygenase) was observed to be up-regulated in all genotypes, most distinctly in genotype NS7774. The protein concentration was thereafter analyzed biochemically, and significant changes were observed among all genotypes under drought-stressed conditions (Figure 11B). A 37% and 23% increment in protein was observed in NS7774 at 5 and 10 days of drought stress, respectively, compared to control. However, reduction was observed more significantly in other genotypes of okra viz., NS7772, Green Gold, and OH3312 under drought-stressed conditions when compared to their respective controls, indicating that they are a sensitive genotype compared to NS7774.

**Figure 12:**
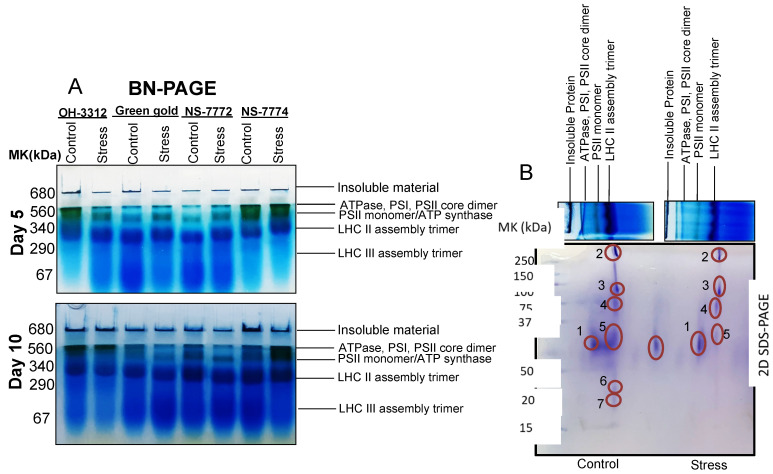
(**A**) First dimension BN-PAGE, as affected by drought stress at 5 and 10 days of interval in okra genotypes NS7774, NS7772, Green Gold, and OH3312, along with respective controls. (**B**) 2D-SDS-PAGE of thylakoid protein complex isolated from NS7774 exposed to drought stress for day 10 along with the control samples. For first dimension BN-PAGE, fresh thylakoid membranes of all okra genotypes were solubilized in 1% BDM at chlorophyll concentration of 1µg·µL^−1^, and the protein sample was separated by 7–12.5% gradient BN-PAGE. For second dimension, gels slices were horizontally laid on top of 12.5% SDS-PAGE and stained with a commercial Coomassie Brilliant Blue R-250 (Bio-Rad, Hercules, CA, USA). Protein identification was based on previous reports and confirmed by MALDI-TOF/TOF-MS, shown in Table 1.

For the thylakoid proteome, blue native page (BN-PAGE) was performed to separate the multi protein complex in its native state from the thylakoids of the chloroplast (Figure 12). The expression of the first protein bands at 680–560 KDa was recognized as PSI, PSII-core dimer and ATP synthase (band 1). The expression level of these protein bands decreased under drought stress conditions for day 5 and day 10among all genotypes of okra compared to their respective control, except for the NS7774 genotype. In NS7774, the expression of PSI, PSII-core dimer and ATP synthase was observed to be almost similar to its control at the initial stage of drought stress and thereafter up-regulated in later stages of drought stress, thus suggesting it to be a resilient rootstock. The second band was identified as PSII-core monomer/Cyt b6/f at 340 kDa and appeared as dark green in colour for all the genotypes. The expression of this band was a little faint for all the okra genotypes under drought stress compared to their respective controls, except for NS7774 genotype, which showed a little high intensity expression of this second protein band. The expression of the last two bands identified as light harvesting complexes (LHC II trimer and LHC III monomer) was almost the same for all the genotypes under drought stress. This band was expressed at a slightly higher level in the genotype NS7774, indicating that it may be a tolerant genotype for drought stress.

From first dimensional BN-PAGE, it was observed that genotype NS7774 could act as a resilient rootstock due to its tolerance towards drought stress, and was thus selected for further second dimension. The 1D BN-PAGE gel lanes were excised and loaded onto SDS-PAGE for second dimensional analysis to identify further complexes of the proteins. We detected seven proteins on 2D-SDS-BNPAGE for the genotype NS7774 under day 10 of drought treatment; it was observed that the number of proteins detected was less for drought stress treatment as compared to its control. (Figure 12B). The identified seven protein profile obtained from our above result was further identified and confirmed by comparing the results with other 2D-BN-PAGE maps of other species by mass spectrometry. The details of identified proteins are given in Table 1.

**Figure 13:**
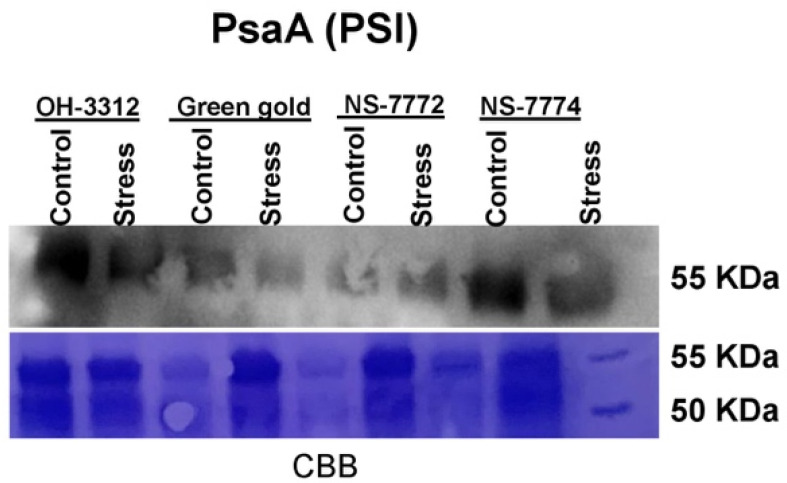
Western blot of photosynthetic protein PsaA, as affected by drought stress at 5 and 10 days of interval in okra genotypes NS7774, NS7772, Green Gold, and OH3312, along with respective controls. In each lane, approximately 50 µg of sample was loaded for each cultivar.

Because PsaA is a core protein of photosystem I, we observed its expression using the western blotting technique (Figure 13) for only the earlier stages of drought stress. The expression of PsaA was significantly reduced in all genotypes of okra under drought stress compared to their respective controls, except a little variation was observed in genotype NS7774. The results of PsaA again signified that NS7774 is a tolerant genotype compared to other selected genotypes.

## 4. Discussion

Drought stress is one of the major abiotic stresses responsible for the reduction of crop productivity [40,41,42,43], plant growth, physiology, and its metabolism [42]. Although a detailed study has been conducted on several crops under drought stress, limited studies are observed on important and underutilized vegetable crops regarding their stress resilience. Therefore, a detailed study on physiological modulations and chloroplast proteome of okra was studied using different genotypes, NS7774, NS7772, Green Gold and OH3312, to identify the resilient rootstock and underlying molecular mechanism. In the present study, a significant change in morphological parameters was observed in all genotypes of okra under drought stress (Figure 1 and Figure 2). The very first symptom noticed in all genotypes was reduced biomass, as observed in several plants from earlier studies [44]. According to the study performed by Greenway and Munns, 1980 [45], the decrease in biomass was also due to decreased water potential in plants cells under drought stress condition, leading to a loss in cell turgidity, which reduced the overall cell elongation and cell division activity in plants. The differences in the genetic makeup of each okra genotype, and the climatic factors, were responsible for the difference in biomass (root and shoot length, and root and shoot weight) of all the four okra genotypes [46].

Because drought stress severely affects water absorption, and in our studies, leaf relative water content was another parameter, we observed its reduction in all the okra genotypes under drought stress. This was another criterion to identify and select a drought tolerant okra genotype as previously studied by a few scientists [47,48]. From our study, it was observed that the okra genotype NS7774 had a minimum reduction in RWC, indicating clearly that it was a drought tolerant genotype and could be considered and used as a resilient rootstock to produce more yield at field level under drought conditions. The osmolyte accumulation under drought stress is another defense mechanism strategy in order to combat water deficiency [49], which shows that NS7774 have higher osmolytes, such as proline, which help the plant to maintain cell turgidity by absorbing more water through roots. This was a clear proof of drought stress and confirmed the sensitivity towards drought stress, as reduced RWC decreased the cell turgor pressure while limiting the water availability for cell expansion process [50,51] (Figure 3A). Drought stress also leads to the production of reactive oxygen species (ROS), including hydroxyl radicals, superoxide, hydrogen peroxide, singlet oxygen and alkoxy radicals, which leads to oxidative damage in plant cells by impairing the normal functioning of plant cell organelles, especially the chloroplast where photosynthesis occurs [52,53,54]. Under prolonged drought stress, oxidative damage increases the MDA content, H_2_O_2_ and O_2_^−^ radicle, thereby damaging the permeability of the cell membrane to a severe level [55]. The lipid peroxidation increase is due to compounds such as superoxide radicals (O_2_^−^), hydrogen peroxide (H_2_O_2_), and hydroxyl radicals (OH) in chloroplasts. In our study, it has been observed that under drought stress, MDA content and oxidative stress markers increased significantly for all okra genotypes (Figure 3B and Figure 4). However, specifically for the genotype NS7774, the increment was less, indicating it to be a tolerant genotype, whereas others were drought sensitive. ROS production under drought stress has previously been observed in different okra genotypes [56,57,58]. In a previous study [59,60,61], the investigators showed that MDA levels increased, especially in the susceptible phenotypes, depending on drought stress, and this increase was related to ROS formation. These results may be imputed to varieties in their genotypic ability to scavenge ROS, to be protected against their oxidative properties, or a combination of both. Our findings led to the hypothesis that drought stress resulted in proteolytic damage, which can induce oxidative stress. During the study, an increase in proline content was observed for the okra genotypes under drought stress, indicating that this is an important parameter for studying the plant’s response (Figure 3C). Differences in the proline accumulation for the okra genotypes are correlated with more tolerance to drought stress, as the tolerant genotype had a higher proline content to maintain its osmotic balance in the plant cells and improve stress tolerance [62]. Our results correlate with many studies previously conducted, which have proved that the production of osmolytes is directly associated with stress tolerance [63,64].

Catalase (CAT) activity and ascorbate peroxidase (APX) activity were determined for all four okra genotypes under drought stress with respect to their control plants (Figure 5). The results showed that CAT and APX activities increased under stress conditions in all genotypes compared to their respective controls. The highest enzyme activities (CAT and APX) were determined for the genotype NS7774 under drought stress. Increased antioxidant activity is one of the defense strategies used by the tolerant okra genotype under stress situations, which helps in scavenging hydrogen peroxide to water in chloroplasts, thereby detoxifying them [65]. Various studies were conducted that generated the same results obtained above for the APX activity under drought stress, where its activity increased in Mongolian milkvetch [66], tomatoes [67], and almonds [68]. Therefore, the production of antioxidant activity is crucial to maintain the equilibrium between the production and scavenging of free radicles [69].

Water transport activity is the major physiological aspect to be modified in crops by abiotic stresses, particularly drought stress. The vascular activity in our results showed an interesting observation among all genotypes of okra (Figure 6). The initial observations under drought stress showed that all okra genotypes recovered from the water stress conditions, while at later stages they declined to a severe level, except for the NS7774 genotype. Due to changes in vascular activity, the other physiological aspect which is severely affected is photosynthesis. As shown in Figure 7, drought stress significantly inhibited the photosynthetic parameters, including photosynthetic rate, transpiration rate, stomatal conductance, and quantum yield of PSII (Fv/Fm) to drought stress treatment. However, it was observed that among the four genotypes, the NS7774 genotype showed the minimum decline in all the photosynthetic parameters for day 5 stress conditions. While at day 10, the same genotype had an increased photosynthetic rate. There could be many reasons behind the decrease, including early leaf senescence, reduced leaf expansion, and reduced photosynthetic pigments [70]. As a result, NS7774 showed better adaptability to drought stress than NS7772, Green Gold and OH 3312, as manifested by their drought response in growth. Chlorophyll fluorescence was measured to calculate the maximum photochemical efficiency of PSII (Fv/Fm) under drought stress [71]. In our study, Fv/Fm decreased with prolonged water stress, and the values in the okra genotype NS7774 was higher than the other okra genotypes under drought stress conditions. This indicates that water stress led to a decrease in the opening of the PSII reaction center, the conversion efficiency of light and the energy used for carbon assimilation accumulation [72]. In our study, drought stress significantly decreased the chlorophyll pigment content in okra genotypes, although this decrease was less for NS7774 (Figure 8A). The reason for this decline could be impairment in the photosynthetic apparatus due to reduction in the parameters mentioned above (photosynthetic rate, stomata conductance, and transpiration rate). Reduction in the chlorophyll pigment due to photo oxidation minimizes the chlorophyll content in the cells, which can cause oxidative stress in plants [73,74]. Similar results were observed for carotenoid pigment for day 5 and day 10 of drought stress (Figure 8B). However, under day 10 of drought stress, a significant increase in carotenoid was observed for the genotype NS7774, indicating its stress tolerance activity, as carotenoid act as an antioxidant agent and helps protect photosynthetic pigments under drought stress [75]. However, the reduction in these pigments is solely responsible for reduced photosynthesis, which ultimately alters stomatal behavior [76,77]. However, in our current study, it was observed that the stomatal density decreased under drought stress, which has a negative impact on photosynthetic parameters (Figure 9). Additionally, from Figure 10, it was clearly observed that under drought stress, all the stomata were closed, and some were partially closed, compared to the control. Therefore, it resulted in a reduced number of chlorophyll molecules under drought stress, which also contributed to a decline in photosynthetic parameters. A similar study was observed for oilseed rape [25], where it was studied that the stomatal index is directly proportional to photosynthesis and its apparatus.

Photosynthesis is a critical and major metabolic process and is prone to any abiotic stress, including drought [8,13,14]. The decline of photosynthesis has been observed due to reduced leaf expansion, improper functioning of photosynthesis machinery, and leaf senescence [13]. To observe the photosynthetic machinery in okra at a molecular level, chloroplast proteomics was the best tool to analyze it. Chloroplast proteome analysis, including traditional SDS-PAGE gel electrophoresis results, showed a determined reduction of RuBisCO protein for all okra genotypes under drought stress, except for NS7774. One of the reasons for the decline in RuBisCo protein could be due to reduced chlorophyll content and lowered photosynthesis (as observed in our results, please see Figure 7 and Figure 8). It can also be due to changes caused in the chloroplast structure due to oxidative stress and reduced protein synthesis [31], as observed in our studies too (Figure 12 and Figure 13). This could further affect the photosynthetic apparatus, including the photosystem PSI. For the genotype NS7774, the expression of this protein was higher, estimating it to be a drought tolerant genotype. The above result was confirmed biochemically, where drought stress reduced the total protein content of the okra genotypes (Figure 11B). According to Khan and Rabb [78], this decline could be due to lowered RWC as also observed in our results (Figure 3A). Production of ROS could also reduce the protein content by causing oxidative stress to the cells, thereby denaturing the protein structure and content. Irrespective of other studies, from our study we observed that NS7774 had the maximum protein content and, in fact, under drought stress its content increased as compared to other genotypes. In our study, we observed reduced chlorophyll content in drought stress for all the genotypes, which resulted in a reduction of chloroplast proteins. Many studies have reported on the changes in the chloroplast proteome under drought stress, such as in rice [79], Arabidopsis [80], and tomatoes [81]. However, before this point in time, very few studies have been conducted on vegetables, particularly okra (an underutilized crop) and the alterations in chloroplast proteins under drought stress using proteomics. The present study also included BN-PAGE to study the expression of thylakoid protein complexes (Figure 12A). It was observed that the multi-protein content decreased for all the genotypes under drought stress. Differences in the amount of different protein complexes varied for different okra genotypes, particularly the amount of PSI and PSII for drought-stressed genotypes. On the one hand, the genotype NS7774 had a greater content of PSI and PSII, indicating it to be a tolerant genotype under extreme drought stress. From previous studies [82], it can be hypothesized that even under high drought stress condition, this genotype, i.e., NS7774, was able to maintain the chloroplast integrity and the photosynthetic pigment content, thereby regulating the electron transport activity under drought stress. A very insignificant change was observed for LHC under drought stress. It is of great importance to note that NS7774 had more expression of the protein complex than other genotypes, which was consistent with the former studies [82]. A total of seven protein spots were identified using a mass spectrometer from 2D BNPAGE for the genotype NS7774 (Figure 12B). These proteins are mainly involved in the photosynthesis process. Compared with the control group, there was little down-regulation in the proteins under drought stress. Although the majority of studies have reported on the morpho-physiological and biochemical activities of the okra plants under drought stress [83,84,85,86], this was the first attempt to report on the sensitivity of photosystems in okra plants under drought stress. In order to confirm our chloroplast proteomic results, western blot was followed up after BN-PAGE to study the expression of photosystem PsaA (Figure 13). Our results showed that the expression of PsaA was reduced under day 5 drought stress for all the okra genotypes except for NS7774, where abundant expression of PsaA was observed in comparison to the other genotypes for the control as well as stress treatment. Overall, from our all experiments on different genotypes of okra it was observed that NS7774 appeared to be drought tolerant while the rest of the genotypes, viz., NS7772, Green-gold, and OH-3312, were observed to be sensitive to drought stress.

## 5. Conclusions

In the present study, a comparative study was conducted for four different okra genotypes, which are NS7774, NS7772, Green Gold and OH 3312, under drought stress conditions, to observe for sensitive and resilient rootstock. The output of this research could assist farmers and other growers in growing such vegetables as okra under drought stress situations, which is an existing problem worldwide, and will provide guidance about the importance of selecting drought tolerant okra rootstocks to overcome this issue and increase crop productivity. Among all the genotypes, NS7774 showed better adaptability towards drought stress than other genotypes, as proved by the above results, and can be used as a rootstock to enhance okra’s drought-tolerance in water deficit regions. However, research is still lacking regarding the comparative analysis of okra genotypes for choosing the drought tolerant genotype and how this tolerant rootstock will aid in combating such abiotic stress situations. Therefore, we conclude, from our present research work that with increasing climate change globally due to environmental factors, and especially drought stress, the growth of plant and yield significantly reduces with time. Hence, we can hypothesize that the use of drought tolerant okra genotypes obtained from the above results will have the potential to preserve the okra yield in the future.

## Figures and Tables

**Table 1:** Proteins identified in 2D-BN-SDS-PAGE map of okra thylakoids.

**Spot No.**	**Protein Name**	**Plant Species**	**Accession Number**	**Protein Score**	**Biological Function**	**Mr Value**	**Calcu. *pI*/Exp. *pI***	**Sequence Coverage**
1	Chlorophyll a-b binding protein	*Capsicum baccatum*	A0A2G2XDV1	78	Photosynthesis	28,213	5.1/4.0	36
2	Photosystem I P700 chlorophyll a apoprotein	*Lupinus angustifolius*	A0A394D0H9	166	Photosynthesis	163,602	6.7/5.0	10
3	Photosystem I P700 chlorophyll a apoprotein	*Lupinus angustifolius*	A0A394D0H9	130	Photosynthesis	163,602	6.7/5.0	13
4	ATP synthase gamma chain	*Desulforudis audaxviator*	ATPG_DESAP	30	Photosynthesis	33,245	9.6/5.0	38
5	CDP-4-dehydro-6-deoxyglucose reductase	*Pseudomonas*	A0A560PPL8	59	Starch and Sucrose Metabolism	36,242	5.57/5.0	13
6	Photosystem Q(B) protein	*Glycine tomentella*	R9ZRU2	40	Photosynthesis	38,866	5.1/5.0	23
7	Cytochrome b6	*Citrus sinensis*	Q09ME7	170	Photosynthesis	24,277	8.89/5.0	33

## Data Availability

Not applicable.
